# Survival from cancer of the oesophagus in England and Wales up to 2001

**DOI:** 10.1038/sj.bjc.6604573

**Published:** 2008-09-23

**Authors:** S Rao, D Cunningham

**Affiliations:** 1 Department of Medicine, Royal Marsden Hospital, Downs Road, Sutton, Surrey SM2 5PT, UK

Most patients with oesophageal cancer have locally advanced unresectable or metastatic disease before symptoms occur. They may present with difficulty in swallowing, indigestion, weight loss, anorexia or occasionally pain on swallowing. The mainstay of diagnosis is by endoscopy and biopsy. Computed tomography scans of the chest, abdomen and pelvis are usually performed to complete staging. In the last decade, endoscopic ultrasonography (EUS) has assumed a central role in the initial anatomic staging of oesophageal cancer because of its accuracy in assessing the extent of locoregional disease (Vilgrain *et al*, 1990; Botet *et al*, 1991; Rosch *et al*, 1992). However, EUS was unlikely to have been widely used before 2001 and thus any impact on survival from better case selection will not be apparent in the data published by Mitry *et al* (2008).

During the period of this analysis, surgery was considered the treatment of choice for localised adenocarcinoma in England and Wales and for those patients who underwent curative resection the median survival was 25 months. In 2002, however, a large randomised Medical Research Council (MRC Oesophageal Cancer Working Group, 2002) study reported a survival benefit for neoadjuvant chemotherapy plus surgery compared with surgery alone. This trial randomly allocated 802 previously untreated patients to two cycles of 5FU/cisplatin preoperatively followed by surgical resection *vs* surgery alone. The 2-year survival was 43% compared with 34% respectively (*P*=0.004). These data changed clinical practice in this country and preoperative chemotherapy with 5FU/cisplatin has become the standard of care for potentially operable oesophageal adenocarcinoma. Chemoradiation is the therapy of choice for patients who are deemed unfit for surgery but whose staging shows localised squamous cell carcinoma or adenocarcinoma.

For advanced oesophageal tumours, there was a shift in the treatment paradigm in the 1990s when several studies demonstrated a survival advantage for chemotherapy over best supportive care. A number of combination chemotherapy regimens were then evaluated in this setting, and epirubicin, cisplatin and infused 5FU (ECF) demonstrated superior efficacy, (median overall survival (OS) of 9.4 months) and quality of life in several randomised trials (Webb *et al*, 1997; Waters *et al*, 1999; Ross *et al*, 2002). Epirubicin, cisplatin and infused 5FU became the regimen of choice for advanced oesophageal cancer in the UK from 1999 onwards.

A national study of 1002 patients evaluated the substitution of capecitabine for infused 5FU and oxaliplatin for cisplatin *vs* the original ECF regimen in patients with previously untreated advanced oesophago-gastric cancer. Capecitabine and oxaliplatin were as effective as 5FU and cisplatin. Furthermore, OS was longer with epirubicin, oxaliplatin and capecitabine (EOX) than ECF (median OS of 11.4 months) with a hazard ratio for death for EOX of 0.80, 95% CI: 0.66–0.97; (*P*=0.02) (Cunningham *et al*, 2008).

Relative 1-year survival increased significantly between the late 1980s and 1990s.This is likely to be partly because of a decrease in postoperative mortality rates. Jamieson *et al* reviewed postoperative mortality rates between 1990 and 2000 and reported an overall mortality rate of 6.7%, which is lower than earlier decades. This rate has continued to fall and the acceptable value is now less than 5% although the postoperative mortality rate for the surgery alone arm in the MRC study was 9% (Jamieson *et al*, 2004). It is now recognised that the outcome after oesophagectomy is strongly related to the institutional volume (number of cases resected per annum) and thus surgical centres have been established nationally (Birkmeyer *et al*, 2002; Dimick *et al*, 2003).

The change in survival may also be attributable to the implementation of chemotherapy for advanced disease towards the late 1990s. There is an increase in 5-year survival during this time period for men although there is no change in this rate for women. This disparity between genders remains unexplained.

The deprivation gap is more marked for 1-year survival compared with 5- and 10-year survival. This may suggest that this effect is not related to disease-specific factors. There is also a widening of the deprivation gap for 5- and 10-year survival over time suggesting that survival has risen more in the affluent than the deprived.

Predicted survival rates from period analysis suggest a continuing small increase in survival. These projections are consistent with current progress in the management of this disease. Endoscopic ultrasonography has become widely adopted for staging of localised disease and FDG PET is being studied to identify occult metastatic disease. Preoperative chemotherapy has demonstrated a significant survival benefit and surgery is being performed at high-volume institutions. The median OS for advanced disease has been prolonged by chemotherapy and with the advent of newer targeted therapies; this is likely to increase further.

Since the late 1990s multidisciplinary teams have been established to ensure the optimal staging, diagnosis and management of oesophageal cancer. The incidence of adenocarcinoma of the oesophagus is increasing and since Barrett's oesophagus has been identified as the main risk factor, research efforts are focusing on intervention and surveillance programmes for this indication. Other molecular and genetic studies are also underway to try and identify prognostic factors that may correlate with clinical outcome.
